# Soft robotics and functional electrical stimulation advances for restoring hand function in people with SCI: a narrative review, clinical guidelines and future directions

**DOI:** 10.1186/s12984-022-01043-1

**Published:** 2022-06-30

**Authors:** Lucas R. L. Cardoso, Vanesa Bochkezanian, Arturo Forner-Cordero, Alejandro Melendez-Calderon, Antonio P. L. Bo

**Affiliations:** 1 Biomedical Engineering, School of Information Technology and Electrical Engineering, The University of Queensland, Brisbane, Australia; 2 College of Health Sciences, School of Health, Medical and Applied Sciences, Central Queensland University, North Rockhampton, Australia; 3 Biomechatronics Laboratory, Escola Politecnica, University of São Paulo, São Paulo, Brazil; 4 School of Health and Rehabilitation Sciences, The University of Queensland, Brisbane, Australia; 5 Jamieson Trauma Institute, Royal Brisbane and Women’s Hospital, Metro North Hospital and Health Service, Brisbane, Australia

**Keywords:** Functional electrical stimulation, Soft robotics, Hands, Fingers, Spinal cord injury, Tetraplegia, Rehabilitation, Therapy, Assistive devices

## Abstract

**Background:**

Recovery of hand function is crucial for the independence of people with spinal cord injury (SCI). Wearable devices based on soft robotics (SR) or functional electrical stimulation (FES) have been employed to assist the recovery of hand function both during activities of daily living (ADLs) and during therapy. However, the implementation of these wearable devices has not been compiled in a review focusing on the functional outcomes they can activate/elicit/stimulate/potentiate. This narrative review aims at providing a guide both for engineers to help in the development of new technologies and for clinicians to serve as clinical guidelines based on the available technology in order to assist and/or recover hand function in people with SCI.

**Methods:**

A literature search was performed in Scopus, Pubmed and IEEE Xplore for articles involving SR devices or FES systems designed for hand therapy or assistance, published since 2010. Only studies that reported functional outcomes from individuals with SCI were selected. The final collections of both groups (SR and FES) were analysed based on the technical aspects and reported functional outcomes.

**Results:**

A total of 37 out of 1101 articles were selected, 12 regarding SR and 25 involving FES devices. Most studies were limited to research prototypes, designed either for assistance or therapy. From an engineering perspective, technological improvements for home-based use such as portability, donning/doffing and the time spent with calibration were identified. From the clinician point of view, the most suitable technical features (e.g., user intent detection) and assessment tools should be determined according to the particular patient condition. A wide range of functional assessment tests were adopted, moreover, most studies used non-standardized tests.

**Conclusion:**

SR and FES wearable devices are promising technologies to support hand function recovery in subjects with SCI. Technical improvements in aspects such as the user intent detection, portability or calibration as well as consistent assessment of functional outcomes were the main identified limitations. These limitations seem to be be preventing the translation into clinical practice of these technological devices created in the laboratory.

## Background

Spinal cord injury (SCI) often leads to motor and sensory deficits, in addition to other complications, such as autonomic dysfunction, respiratory problems and urinary incontinence [1]. Among these complications, one of the major therapeutic priorities of people with tetraplegia is the recovery of arm and hand function since they are essential to independently perform most of the activities of daily living (ADLs) [2–4].

The rehabilitation of arm, hand and finger-related functional abilities after SCI can follow different approaches. One of them is through invasive procedures, like nerve and tendon transfer, in which preserved working nerves (tendons) are surgically re-directed to proximal non-functioning motor pathways [5]. Although this technique has the potential to produce relevant functional outcomes, it may demand long training time for adaptation post-surgery [5].

Another alternative to recover hand function after SCI are activity-based therapies. These comprise several training protocols and techniques, usually delivered under the supervision of physical or occupational therapist, and have the potential to increase range of motion, decrease pain and spasticity or recover lost functional movements, relying on the principles of neuroplasticity [6]. When the patient’s limb is activated, combining volitional control and external assistance, sensory afferent input is produced, which triggers a series of neurorestoration processes (e.g., synapse formation, remyelination, neural reorganization and repair), either in supraspinal or in spinal structures [6–8]. However, due to the high number of repetitions required to enhance neuroplastic adaptations, this type of intervention can be time-consuming and costly [7, 9, 10]. To potentially reduce treatment cost and time, and improve functional outcomes, activity-based therapies can be supported by technological hand neuroprostheses. In addition to therapeutical purposes, these engineering features have been employed as assistive devices, increasing the user’s independence and augmenting the overall practicing time.

Functional electrical stimulation (FES) is one of the technologies used to build neuroprostheses to support activity-based training after SCI. During a conventional FES therapy, subjects are encouraged to voluntary activate their muscles to perform a certain task while the FES system stimulates the muscles using superficial or implanted electrodes [11, 12]. According to this approach, purposeful movements are produced in parallel to a combination of cortical activation (due to the voluntary attempt) and peripheral stimulation. The FES produces additional afferent information thus enhancing the practice-induced brain and spinal plasticity [13–15]. A common method used to trigger electrical stimulation is through a push-button. However, a more intuitive system detects user intent via physiological signals, e.g., electroencephalography (EEG) or electromyography (EMG), which increases usability and learning outcomes, by pairing stimulation with movement intention [16]. Despite promising results as a therapeutical tool [17], FES devices are limited in generating high accuracy control and muscle selectivity [18]. In this respect, implanted systems [19] or superficial multi-pad electrode matrices [20, 21] can yield better outcomes but they still have many obstacles, such as the limitations of its use in case of lower motor neuron damage [22, 23] or in people with cervical injury without any volitional control of the hand. [14].

Robotic systems are also employed to support activity-based therapy for hands after SCI. Typically, these are non-portable devices that are able to assist end-user’s hand in a clinical setting, throughout repeatable and predictable movement patterns [24]. However, most of these devices are bulky and are built using rigid links, which hampers the biomimetics of the human hand [25], and possibly limits the potential outcomes of the therapy [26]. In this sense, neuroprostheses based on Soft Robotics (SR) devices have emerged as a specific category of robotic rehabilitation systems, relying on soft actuators (usually back-drivable) and flexible links, increasing comfort and flexibility to adjust to the contours of the human body [25, 27–29]. SR devices developed for hand function are also intended to be lightweight and portable, possibly for home-rehabilitation use, which is important to increase end-user adherence to treatment and also to meet assistance needs in ADLs. The underlying neuroplastic process associated to the use of SR tools is the same as observed in conventional activity-based therapies, since they also provide mechanical assistance for the movement execution. However, they are intended to increase the user engagement (by supporting activities in a daily basis) and consequently increase the number of repetitions (practice time), for a more affordable cost compared to the constant supervision of a physiatrist [28].

Noticeably, FES and SR have complementary features which encourages protocols combining both technologies. In a recent review, Dunkelberger and colleagues described a hybrid muscle stimulation and robotic assistance that was used for upper limb movement in people with SCI [30]. Even if the review did not focus on hand function or in SR, the authors concluded that the combination of FES and SR was promising, but argued that technological advances (e.g., improve tunability, reduce size and weight or detect user intent in an intuitive and unobtrusive way), both in FES and robotics, should be achieved to be fully integrated in an efficient hybrid system [30].

The present narrative review aims to identify the effects of FES, SR and their combination in the recovery of hand function in people with SCI. Therefore, this review summarizes the most recent research articles that presented any hand functional outcomes in people with SCI, using neuroprostheses based on FES and/or SR, either for assistance or therapy purposes. Results from this review will inform engineers on the next steps to develop these technologies and will allow clinicians to use this information as easy-to-use clinical guidelines.

### Related reviews

There are four aspects from previous reviews on this topic that are worth discussing: the intervention aim, target population, affected function and assessment approach. Table 1 presents some of the related study reviews from the last 5 years.

**Table 1:** Related study reviews from the last 5 years

Group	Intervention aim		Target population		Affected function			Assessment	
	Assistive	Therapeutic	SCI only	Multiple	Hand only	UL	Multiple	P	S
*Soft robotics*									
Tran et al. 2021 [31]	$\checkmark$	$\checkmark$		$\checkmark$	$\checkmark$			$\checkmark$	
Proulx et al. 2020 [28]	$\checkmark$	$\checkmark$		$\checkmark$	$\checkmark$			$\checkmark$	
Dávila-Vilchis et al. 2020 [27]	$\checkmark$	$\checkmark$		$\checkmark$	$\checkmark$				$\checkmark$
Sarac et al. 2019 [32]	$\checkmark$	$\checkmark$		$\checkmark$	$\checkmark$				$\checkmark$
Chu et al. 2018 [25]	$\checkmark$	$\checkmark$		$\checkmark$	$\checkmark$				$\checkmark$
Gassert et al. 2018 [33]		$\checkmark$		$\checkmark$			$\checkmark$	$\checkmark$	
Shahid et al. 2018 [29]	$\checkmark$	$\checkmark$		$\checkmark$	$\checkmark$				$\checkmark$
FES									
Marquez-Chin et al. 2020 [34]	$\checkmark$	$\checkmark$		$\checkmark$			$\checkmark$	$\checkmark$	
Kapadia et al. 2020 [17]		$\checkmark$		$\checkmark$		$\checkmark$		$\checkmark$	
Luo et al. 2020 [35]	$\checkmark$		$\checkmark$				$\checkmark$	$\checkmark$	
Milosevic et al. 2020 [14]	$\checkmark$	$\checkmark$		$\checkmark$		$\checkmark$		$\checkmark$	
Degnan et al. 2017 [36]	$\checkmark$		$\checkmark$			$\checkmark$		$\checkmark$	
FES + Soft Robotics									
Dunkelberger et al. 2020 [30]	$\checkmark$	$\checkmark$	$\checkmark$			$\checkmark$			$\checkmark$
Our study	$\checkmark$	$\checkmark$	$\checkmark$		$\checkmark$			$\checkmark$	

*UL* upper limbs, *P* user-centric, *S* system-centric

*Intervention aim* Devices can be designed to meet therapeutic or assistive needs. One key difference between these types is that assistive devices are usually worn continuously or, at least, during ADLs. On the other hand, the use of therapeutic devices is restricted to short periods of time, such as the duration of each therapy session. However, the main underlying processes involved in the positive outcomes of therapeutic interventions are associated to practice-induced neural plasticity [14, 33]. Therefore, it is worth considering the benefits of hybrid systems that integrate therapeutic and assistance tasks in a single device [27]. Interestingly, according to Table 1, current reviews in SR aimed for both tasks, whereas FES studies usually focus on a single intervention aim. For the purposes of providing guidelines for both clinicians and engineers, this review will focus on studies with both interventions.

*Target population* Although different neurological conditions share similar physical deficits, the mechanisms involved in their rehabilitative process may differ significantly [33]. In particular, people with SCI present specific voluntary muscle activation limitations after injury that require specialized task-oriented rehabilitation [37]. Although SR or FES assistive devices may attend the needs of diverse clinical populations, their everyday performance and long-term improvements in functional outcomes can significantly vary based on level, severity and type of clinical condition [28]. Thus, results from different target population are not strictly interchangeable. To date, as shown in Table 1, there is no literature on SR devices designed for hand function recovery exclusively for people with SCI. There are some studies that include people with SCI together with other conditions [28, 31, 33], but those studies did not present functional outcomes separately, which limits the interpretation of the results and hampers the translation to clinical practice, specifically for people with spinal cord injury.

*Affected function* Some literature reviews were very broad and covered studies with a great variety of functional outcomes, from limbs strength to bladder control (see [35] for a comprehensive review on FES treatment after SCI), while others were more specific including only one therapeutic aim (Table 1). For people with tetraplegia, recovery of arm and hand movements is usually ranked as one of the highest priorities, because these functions have a high potential to restore their daily independence [2–4]. However, although most ADLs depend on arms and hands, the neuroprostheses developed for each of these functions have distinct characteristics and are at different stages of technological development. Specific investigations are required to understand the potential of SR and FES for reaching (e.g., arms and shoulders) or grasping (e.g., hand and fingers) and, according to Table 1, there is not current literature review on FES devices exclusively investigating hand function in people with SCI.

*Assessment approach* Some reviews focused on studies evaluating the technical features of the device, but did not consider the end-user needs or functional outcomes. On the other hand, some studies present and discuss their results in terms of functional outcomes using standard Clinical Outcome Assessments (COA) [38]. Studies with the first approach may be referred as system-centric whereas the second have a user-centric point-of-view [30]. User-centric studies are particularly important for clinicians who want to use SR and FES devices based on end-user’s individual characteristics.

**Fig. 1:**
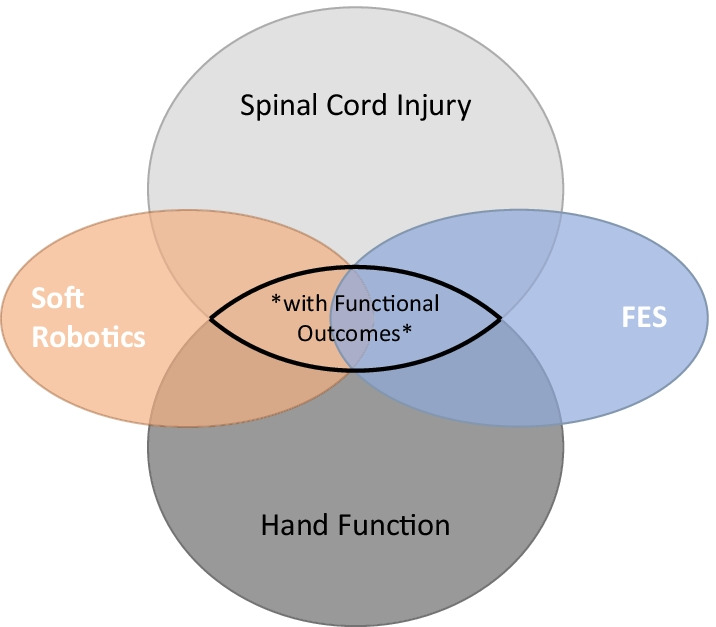
The focus of the present narrative review. Report of functional outcomes is key to determine the role of FES and SR-based neuroprostheses in the hand function recovery after SCI

This work includes the analysis of functional outcomes of FES, SR and their combination in the recovery of the hand function of people with SCI. In this context, the present study aims at providing a guide for engineers to help in the development of new FES- or SR-based hand neuroprostheses, and for clinicians to serve as clinical guidelines in order to assist and/or recover hand function in people with SCI. We intend to fill the gap in the literature identified in Table 1, by focusing on the intersection highlighted in the Venn diagram of Fig. 1.

## FES and SR-based neuroprostheses for hand function after SCI

### Methods

#### Selection process

The selection process of this review was based on the PRISMA method (preferred reporting items for systematic reviews and meta-analyses) [39] and included the following steps:

1. *Identification:* collection of all records identified through the search parameters, in all considered databases. Duplicate studies were removed;
2. *Screening:* first, papers were screened based on the following inclusion criteria: (i) published in English (ii) published since 2010 and (iii) article, journal or conference paper. Reviews of any kind (literature, narrative or systematic) or book chapters were excluded. In the next step, full-texts were screened in terms of the eligibility criteria specified below;
3. *Inclusion:* final collection of studies, including documents that did not appeared in the initial identification.

It is worth noting that this process was repeated twice, first for the SR and then for the FES studies following the searching criteria specified for each of them.

#### Eligibility criteria

The inclusion and exclusion criteria used to narrow the literature search is as follows:

Inclusion criteria:

- Either therapeutic or assistive devices;
- Only subjects with SCI;
- Either therapy or assistance of hands and fingers;
- Novel device or experimental findings using a previously studied system device;
- Functional outcomes—using standardized or non-standardized tests.

Exclusion criteria:

- Studies that included combined therapies—with drugs (e.g., BOTOX), surgery, blood flow restriction, Transcranial Magnetic Stimulation were excluded, except when combined with conventional occupational therapy;
- Other clinical conditions, such as stroke;
- Wrist function only;
- Articles that only report impairment improvements (e.g., range of motion or muscle strength).

#### Search strategy

The literature search was carried out in the following databases: IEEE Xplore, Pubmed and Scopus. The search queries were composed by three basic groups. The first group specified the technology, thus it varied when searching for SR devices (“robotic”, “robot”, “soft”, “wearable”, “exoskeleton”) or FES systems (“electrical stimulation”, “FES”, “NMES”). The other group for both SR and FES was related to the function (“hand”, “finger”, “thumb”, “glove”) and to the clinical condition (“sci”, “spinal cord injury”, “tetraplegia”, “quadriplegia”, “paralysis”, “hand impairment”).

### Results

#### Summary overview

**Fig. 2:**
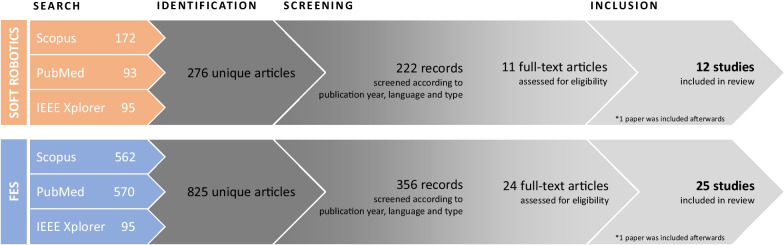
Summary results of the literature search process

The final search for this review was completed in March 2022. After removing duplicate records, a total of 276 and 825 articles, were identified from the SR and FES searches, respectively. In terms of the SR studies, the first part of screening process excluded 54 records and the second part of the screening process (full-text screening) resulted in the exclusion of an additional 211 articles. Regarding FES studies, both procedures excluded 469 and 332 papers, respectively. One additional document from SR [40] and another from FES [41], that did not appear in the first screening phase, were added in the inclusion phase. Thus, the final selection was 12 SR and 25 FES documents. Figure 2 shows a summary of the article selection process.

Several documents were excluded due to the lack of evidence on functional outcomes. In some cases, the article reported outcomes in terms of the user’s range of motion (ROM) or muscle strength [42–45]. These types of measurements represented assessments of impairment, instead of assessment of functional outcomes, thus the documents were excluded from the final selection. Two articles reported the results of the same experiment [11, 46], thus they were analysed as a single study. One [47] was a retrospective study that included the same results reported by [46] and [11], therefore it was excluded. One paper used a device that combined FES and robotic assistance for the fingers [48]. Additionally, one paper combined a FES neuroprostheses and a motorized hand orthosis in the same rehabilitation protocol [49].

The articles included in this review presented and validated FES or SR systems by focusing on a certain intervention aim, either therapeutic or assistive modes. From the 12 articles selected for the SR group, only 1 described a therapeutic protocol [50]. On the other hand, from FES articles, 9 out of 25 presented therapeutic applications.

Interestingly, a few research groups have published three or more articles that were included in this review, often reporting results obtained using different versions of the same device. For example, from the SR collection, three documents [51–53] were from the same laboratory at Harvard University. Three studies [40, 54, 55] were part of to the same laboratory at Seoul National University. In terms of the FES collection, five research studies [20, 21, 56–58] had the same origin (Ohio State University) and shared many of their authors. Other examples were the Case Western Reserve University and the Toronto Rehabilitation Institute (University of Toronto), which were two other research groups with four [19, 59–61] and three articles each [11, 62, 63]. Together, these five research groups were involved in 18 research articles, representing nearly a half of the entire collection.

#### Technical aspects

Tables 2 and 3, presented the main technical characteristics of the devices that were used or developed by each study included in the SR and FES collections, respectively.

The columns Active Fingers, Assisted Motions and Grasp Patterns are associated to the active support provided by the device to the user. In this paper, the words “active” or “passive” always refer to the device, not to the end-user. Thus, an active system necessarily has an actuator, while passive component may represent springs, wires or rigid and sliding structures.

The tables also include the Home-based category, that evaluates whether the device is prepared to be used at home. The devices were classified according to characteristics like portability, ease to don and doff, and weight, as being either “Ready” (R) for in-home use/therapy or “Potentially” (P) prepared (e.g., the device is portable and lightweight, but it was not tested outside the laboratory). In some cases, not enough evidence was presented to determine the classification (“Unclear”, or U).

**Table 2:** Technical aspects of each study included in the SR collection

Study / device used$^{\text{a}}$	Interv. aim$^{\text{b}}$	Actuation type$^{\text{c}}$	User intent detection$^{\text{d}}$	Active fingers	Assisted motion$^{\text{e}}$	Grasp Patterns	Weight [g]		Home- based$^{\text{f}}$
							Main	Other	
Correia et al. 2020 [53]/ no name [RP]	A	Pneumatic	Button	All	F/E	Palmar 3-point	122–149	–	P
Zhou et al. 2019 [52]/ no name [RP]	A	Pneumatic	Pressure Bend sens.	All	F/E	Palmar 3-point	122–149	–	P
Cappello et al. 2018 [51]/ no name [RP]	A	Pneumatic	Button	All	F/E	Palmar 3-point	77	5000	P
Bützer et al. 2021 [64]/ ETHZ RELab tenoexo [RP]	A	TLSS	Button sEMG	All	F/E/ Tb/Td $^{\text{g}}$	Palmar Lateral	148	492	P
Nazari et al. 2021 [65]/ no name [RP]	A	TLSS	sEMG	All	F/E/ Tb/Td	Palmar Lateral	228	–	P
Tran et al. 2020 [66]/ FLEXotendon Glove-II [RP]	A	Cable	Voice cmd.	Thumb Index Middle	F/E/ Tb/Td $^{\text{h}}$	–	297$^{\text{i}}$	–	U
Yoo et al. 2019 [67]/ no name [RP]	A	Cable	sEMG	Thumb Index Middle	F	3-point	190	260	P
Kim et al. 2019 [40]/ Exo-Glove Poly [RP]	A	Cable	Camera	Thumb Index Middle	F/E	3-point	–	1630	U
Kang et al. 2018 [55]/ Exo-Glove Poly II [RP]	A	Cable	Button	Index Middle	F/E	3-point	104	1140	U
Randozzo et al. 2018 [68]/ EMOVO Grasp [C]	A	Cable	App	All$^{j}$	F/E	–	50	930	P
In et al. 2015 [54]/ Exo-Glove [RP]	A	Cable	Bend sens.	Thumb Index Middle	F/E	3-point	194	–	U
Osuagwu et al. 2020 [50]/ SEM Glove [C]	T	Cable	Pressure sens.	Thumb Middle Ring	F	3-point	85	600	R

^a^C: commercial (including products no longer commercialized); RP: research prototype

^b^ Intervention aim. A: assistance; R: therapy

^c^TLSS: three-layered sliding spring mechanism

^d^sEMG: superficial electromyography

^e^F: flexion; E: extension; Tb: thumb abduction; Td: thumb adduction

^f^Prepared for home-based use. R: ready; P: potentially; U: unclear

^g^The thumb adduction and abduction are passive

^h^The finger extension and the thumb abduction are passive

^i^The orthotics weights 69 g and the actuators (attached to the forearm) weights 228 g

^j^Although the authors present the device with all fingers actuated, they tested it using only the thumb, index and medium fingers. The hyphen means lack of information

**Table 3:** Technical aspects of each study included in the FES collection

Study / device used$^{\text{a}}$	Interv. aim$^\text{{b}}$	Actuation type	User intent detection$^{\text{c}}$	Grasp patterns	Stimulation Parameters$^{\text{d}}$				Home- based$^{\text{e}}$
					CH	F [Hz]	W [$\mu$s]	A[mA]	
Fattal et al. 2022 [69]/ STIMEP [RP]	A	Implanted	sEMG Should. pos.	Palmar Lateral	2	25	100–150	0.08–0.58	U
Cajigas et al. 2021 [49]/ Bioness H200 [C]	A	Superficial	iBMI	Palmar Lateral	3	–	–	–	R
Venugopalan et al. 2020 [70]/ TetraGrip [RP]	A	Superficial	Should. pos.	Palmar Lateral	4	–	–	–	U
Müller-Putz et al. 2019 [41]/ TetraGrip [RP]	A	Superficial	Should. pos.	Palmar Lateral	2 30e	16	500	–	R
Annetta et al. 2019 [21] Bockbrader et al. 2019 [20] Colachis et al. 2018 [58] Schwemmer et al. 2018 [57] Bouton et al. 2016 [56]/ BCI-FES [RP]	A	Superficial	iBMI	Palmar Lateral 3-point 2-point	130e	50	500	0–20	P
Heald et al. 2019 [61]/ FreeHand [C]	A	Implanted	sEMG	Palmar Lateral	8	–	–	–	P
Kilgore et al. 2018 [19] Memberg et al. 2014 [59]/ IST-12 [C]	A	Implanted	iEMG	Palmar Lateral	12	12–16	0–255	0–20	R
Ajiboye et al. 2017 [60]/ no name [C]	A	Implanted	iBMI	Palmar Lateral	36	12.5	0–200	20	P
Rohm et al. 2013 [71]/ MotionStim [C]	A	Superficial	sBMI Should. pos.	Palmar Lateral	4	–	–	–	P
Pedrocchi et al. 2013 [72]/ RehaStim [C]	A	Superficial	None$^{f}$	Palmar	–	20	–	–	U
Gan et al. 2012 [73]/ SRS [RP]	A	Implanted	Tooth click	–	3	–	–	–	R
Thorsen et al. 2013 [74]/ MeCFES [RP]	A/T	Superficial	sEMG	–	1	16	300	–	P
Jovanovic et al. 2021 [63]/ Compex Motion [C]	T	Superficial	sBMI	Palmar Lateral	4	40	250	–	U
Scott et al. 2018 [48]/ HandGlove 200 [RP]	T	Superficial	None	None	–	–	–	–	U
Trincado-Alonso et al. 2017 [75]/ INTFES [RP]	T	Superficial	sBMI	–	1	40	350	14–26	U
Harvey et al. 2016 [76]/ ReGrasp [C]	T	Superficial	Tooth click	–	3	50	200	<63	R
Kapadia et al. 2013 [62] Popovic et al. 2011 [11] (or Kapadia et al. 2011 [46])/ Compex Motion [C]	T	Superficial	Button	Lateral 3-point 2-point	4	40	250	8–50	P
Martin et al. 2012 [77]/ Empi 300 PV [C]	T	Superficial	Button	Lateral 3-point	2	30–50	300	20–40	P
Kowalczewski et al. 2011 [78]/ ReGrasp [C] EMS 7500 [C]	T	Superficial	Tooth click None	– –	3 2	– –	– –	– –	R

^a^C: commercial (including products no longer commercialized); RP: research prototype

^b^Intervention aim. A: assistance; R: therapy

^c^sEMG: superficial electromyography; iEMG: impanted electromyography; sBMI: superficial brain machine interface; iBMI: implanted brain machine interface; Should. pos.: shoulder position

^d^CH: number of channels (note: “e” means number of electrodes in case of stimulation matrix); F: frequency; W: pulse width; A: amplitude

^e^ Prepared for home-based use. P: potentially; R: ready; U: unclear

^f^ When tested with people with SCI to control the hand neuroprostheses. The hyphen means lack of information

1) Actuation type

*Soft robotics.* The final SR collection included three types of actuation, namely, pneumatic, cable driven and based on a three-layered sliding spring (TLSS) mechanism. Pneumatic systems [51–53] uses pressurized air to inflate air-tight bladders that form a glove with attachment points. As the fabric compresses or expands it induces finger flexion or extension.

Seven, out of the twelve papers, presented or tested a device actuated by cables [40, 50, 54, 55, 66–68]. Cable driven is a bioinspired actuation system that mimics the tendon mechanism of a human finger. This system drives finger flexion or extension by tensing cables (typically, using Bowden cables) guided throughout the fingers and attached to the distal phalanges using fabric straps.

In the Exo-Glove Poly [40] and in the Exo-Glove Poly II [55]—the second and third generation of In and colleagues’ work [54]—straps material was replaced by waterproof polymer, aiming to increase hygiene and comfort aspects. The hand orthosis presented by Yoo and colleagues [67] was designed to enhance tenodesis grip, and it was based on 3D-printed components in order to become more affordable and customizable. Different from the other systems driven by cables, the “mano” device [68] uses Bowden cables in a dual manner: as artificial tendons and as structural elements. In this device, with Bowden cable sheaths attached only to the dorsal side of the hand and finger phalanges, the palm and fingertips were left fully uncovered, increasing the users’ opportunity to experience any sensation on their hands.

Two studies [64, 65] presented devices based on a three-layered sliding spring mechanism, adapted from the concept originally presented in [79]. In TLSS, two spring blades are layered, with rigid elements connecting them. By moving one sliding spring and fixing the other, the relative length of the springs changes, bending the springs in specific locations, mimetizing the human finger. In [64], the sliding springs are actuated by Bowden-cables, that transmit the torque of electric motors storage in a backpack. In [65], the electric motors are directly attached to the TLSS. Both hand exoskeletons based on TLSS are mounted on the dorsal side of the hand, which is advantageous for somatosensation aspects.

The “mano” device and the SEM Glove (Bioservo Technologies AB), tested by Osuagwu and colleagues [50], are the only systems that are currently commercially available.

*FES.* Two main modes to delivery stimulation were identified, either by using superficial [11, 20, 21, 41, 48, 49, 56–58, 62, 63, 70–72, 74–78] or implanted [19, 59–61, 69, 73] electrodes.

Six out of twenty five articles described experiments using implanted electrodes for FES, and four of them came from the same research laboratory [19, 59–61]. The FreeHand system [61] and its latest version, the IST-12 [19, 59], were fully implantable, while Ajiboye and colleagues [60] used another system with percutaneous (“readily removable”, according to the authors) electrodes. In [69], the authors used a pair of multi-contact cuff electrodes, which is intended to increase the selectivity during functional movements.

All the implanted FES systems used an electrical current with a biphasic waveform. Only two studies reported on the frequency (up to 25 Hz) and pulse width (maximum 255 $\mu s$).

Among studies involving superficial stimulation, the number of channels (and electrodes) varied from a single channel [74, 75] to multi-pad systems, using an array of multiple electrodes [20, 21, 56–58]. The strap-based matrix system composed by 130 electrodes was employed by five studies, all of them from the same research laboratory [20, 21, 56–58]. One study designed a stimulation setup combining two channels with a pair of electrodes (for finger extension and thumb/finger flexion) and one array with 30 pads (for thumb extensor/opposition) [41]. In [72] a multi-pad system was also used, but the authors did not mention the number of electrodes.

The stimulation parameters of the superficial systems included pulse frequencies ranging from 16 to 50 Hz and amplitudes up to 63 mA. The strap-based matrix system was the only with a monophasic current waveform.

2) User intent detection

*Soft robotics.* Six different methods for user intent detection were identified in the final SR collection. The most frequent method employed is the push button, representing 4 out of 12 papers (33$\%$) [51, 53, 55, 64]. This mechanism is very simple: when the button is pressed, actuators close or open the hand. The “mano” device [68] uses a similar approach, with a user interface (smartphone app) in which the subject (or the therapist) is able to individually adjust the angle of each finger.

Pressure (or force) sensors were used by other two devices [50, 52]: in Zhou’s work [52], a state machine controller determined the hand pose based on the signals of pressure sensors placed on the palm and fingertips; and in the SEM Glove [50] proportional pulling force in the flexion direction is applied according to what is sensed from fingertips’ pressure sensors. Within the final SR collection, other user intent detection methods were identified: wrist extension, based on the signals of a bending sensor positioned on the dorsal side of the wrist [54]; myoelectric (EMG) signals, recorded from either the ipsilateral biceps or the upper trapezius muscle [67], or other muscles [64, 65]; voice command, in which the user is able to combine keywords to determine the action to be performed, creating a customized dictionary [66]. In [40], Kim and colleagues used a first-person-view camera to record spatial/temporal information and used it to detect user intent. The camera approach is particularly interesting because it detects user’s intention without requiring any previous calibration or initialization.

*FES.* Three studies used a push button to trigger electrical stimulation [11, 62, 77]. The ReGrasp (Rehabtronics) [76, 78] and the system presented by [73] have a “behind-the-ear bluetooth device” that senses the user’s tooth click, through which stimulation is activated. Compared to the push button method, the tooth click has the benefit of letting both hands free, thus it is more suitable for assistive devices.

Other studies employed EMG signals to detect muscular activity, using either superficial [74] or implanted electrodes [19, 59, 61, 69]. Fattal and colleagues also tested the detection of movements through the user’s shoulder position using a inertial measurement unit (IMU) [69]. Shoulder control was also implemented by other studies [41, 70, 71].

Another approach for user intent detection is through the direct communication between the brain and the stimulation system, which is called Brain Machine Interface (BMI). Trincado-Alonso and colleagues [75] developed a non-invasive BMI based on superficial EEG recordings. Their therapeutic platform classifies the EEG signals and triggers the FES when motor attempt is detected. The user interacts with a visual interface. In [63] the BMI is also superficial, but the authors used a single EEG channel per hand. Differently, the BMI developed by the Ohio State University group [20, 21, 56–58] or presented by [60] consists of a microelectrode array implanted in the brain motor cortex of a subject. Instead of intracortical but still implanted, Cajigas and colleagues recorded electrocorticographic (ECoG) signals from the brain surface [49].

There are some systems that do not rely on any method to detect user intent [48, 78]. These were therapeutic devices that cyclically delivered electrical stimulation during a predetermined period, together with physical and occupational therapy [78] or repetitive task with robotic assistance [48]. The MUNDUS project, the system presented in [72], can detect user intention of movement through multiple ways, but none of them were tested with people with SCI to control the hand neuroprostheses.

3) Active support

*Soft robotics.* In terms of the assisted motion, most devices actively support flexion and extension (i.e., hand opening), except the SEM Glove [50] and the Yoo’s [67] device that are restricted to flexion. The FLEXotendon Glove-II [66] produces finger flexion and thumb abduction actively, but the hand opening movement (finger extension and thumb adduction) is passively actuated using a flexible wire attached to the dorsal side of the hand. The ETHZ RELab tenoexo [64] also uses a passive structure to produce thumb abduction and adduction.

Regarding finger assistance, the pneumatic gloves [51–53] and the devices based on TLSS mechanism [64, 65], actuate all the four fingers and thumb, although the three-fingered grasp was the most frequent configuration observed in the final collection. The combination of thumb, index and middle fingers is used by four devices [40, 54, 66, 67], while the configuration of thumb, middle and ring fingers is adopted by the SEM Glove, [50]. The “mano” device [68] is reported in the study as being able to actively assist the four fingers and the thumb, however, during the described experimental setup, the three-fingered configuration was used. The Exo-Glove Poly II [55] is the one with less finger support (only the index and middle fingers), because a passive structure is used for the thumb (i.e., without an actuator). This structure keeps the thumb in opposed position (adduction), which helps with manipulation of objects.

Most devices are prepared to produce a 3-point grasp type (with different finger configuration) a precision grasp according to Cutkosky’s taxonomy [80]. In the pneumatic gloves [51–53] and in the devices based on TLSS mechanism [64, 65] a power grasp (according to Cutkosky’s taxonomy) can be generated too since all fingers are actuated. However, in [64] and [65], the fingers are unable to move independently. Based on the description provided in Randazo’s study [68], “mano” system is not prepared to support specific hand positions, but the angles of the fingers can be individually adjusted as per the user’s needs. The same characteristic was observed in FLEXotendon Glove-II [66], in which the voice commands can be customized by the user to produce specific grasp patterns or sequence of movements.

*FES.* The grasp patterns produced by a FES system depend on the number of electrodes, because with more channels the device is able to stimulate more muscles, thus eliciting more hand positions. With the 1-channel devices [74, 75], hand closing was the only possible hand movement, and there was no information about the grasping type. However, in [20, 21, 56–58] a multi-pad system was reported, reaching higher resolution and producing up to seven grasp patterns that could be grouped in precision grasps of thumb plus 2 or 3 fingers, and power grasps, like palmar and lateral key. In general, most devices were programmed to generate two hand positions, which were, palmar and lateral grasps. In [48], the device was not able to produce a functional grasp, but can reproduce repetitive finger movements. The system presented by Gan and colleagues is described by generating hand opening and closing, but the authors did not mention a specific grasp pattern [73].

4) Home-based use

*Soft robotics.* The use of lightweight systems (including the orthotic component and the control box) is important to provide users with at-home assistance or therapeutic activities for prolonged periods of time. In this respect, devices range from 50g [68] to 228g [65], when considered only the part that is attached to the hand. The FLEXotendon Glove-II [66] is the heaviest system if the motors (attached to the forearm) are considered (69g of the orthotic components plus 228g of the actuators). Cappello and colleagues [51] presented a 5 kg-control box (that some users reported to be noisy), designed to be mounted on a wheelchair or placed on a table. The “mano” device [68] includes a chest-pack that weights 930g and hosts the actuators, energy storage and control units. SEM Glove [50] and ETHZ RELab tenoexo [64] are two systems reported as being fully portable.

Another desirable feature for at-home therapy is the ease to independently don and doff the device. In most studies, the authors did not mention whether the subjects were able to don and doff the system without help. Osuagwu and colleagues [50] informed that the majority of participants were able to independently don the glove, although the participants with more severe hand impairment required a carer’s assistance. In [64], the unique subject that experienced the system could don (spent 3.5 minutes) and doff (less than 30 seconds) the ETHZ RELab tenoexo without any help, but the authors did not mention whether he was familiar with the device before the test.

Although all devices of the final SR collection were reported as being portable, only in one part of the studies the experimental setup was carried at the participant’s home. This fact is the only evidence that shows the system is most likely prepared to be taken away from the laboratory. That was the reason why the studies of [51, 67, 68] [65] and [64] were classified as “potentially” prepared for home-based use or therapy. The articles [53] and [52] fell in the same classification because they tested the latest version of Cappello’s [51]. Osuagwu’s study [50] was the only one classified as “ready”, because it focused on home-based therapy, describing a self-administered protocol specifically designed for this purpose.

*FES.* Most FES studies used commercially available portable devices, such as, the implanted FreeHand [61] or the IST-12 [19, 59], the Compex Motion [11, 62], the MeCFES [74], the Empi 300 PV [77] and the ReGrasp [76, 78]. However, only a small part of these studies performed experiments at the end-user’s home, like in [41, 59] and particularly in [78], in which home tele-rehabilitation was tested. Other studies were not clear about the system portability [48, 63, 69, 70, 72, 75].

In regards to the FES system with an invasive BMI, the authors in [20, 21, 56–58] mentioned the portability as a limitation, thus preventing it to be promptly tested at home. In [49], although some trials were performed in the subject’s home, only a motorized hand orthosis was tested in this environment and no functional assessments were done in home setting (due to the COVID-19 pandemic).

In terms of ease to don and doff, most superficial systems depend on placing self-adhesive electrode pads on the skin, which is time consuming and usually require support [11, 62, 71, 74, 75, 77, 78]. The ReGrasp device, used by [76, 78], incorporated the electrodes into a custom-made garment [76, 78] that facilitated donning and doffing and ensure repetitive electrodes placement. The implanted systems had the advantage of not requiring an additional set-up.

#### Functional outcomes

Eleven different standardized functional assessment tools were identified from the SR and FES collections. Furthermore, most studies also evaluated end-users using non-standardized tests [21, 40, 54–56, 59, 60, 65, 68, 71–73].

Table 4 shows, for each study, details about the population (sample size, lesion completeness and time since injury), and the assessment tools adopted by each one. The arrows were used to indicate positive or negative outcomes, only when statistical analysis was presented.

**Table 4:** Functional assessment tools and population details

Study	Intervention aim	Population$^{a}$			TRI-HFT	JTHFT	FIM	SCIM	BBT	GRT	GRASSP	ARAT	CUE-T	QIF-SF	MCS	nSTD
		N	LC	TSI												
*Soft robotics*																
Osuagwu et al. 2020 [50]	Therapy	15	I	Ch	$\uparrow$											
Correia et al. 2020 [53]	Assistance	13	–	Sb/Ch		$\downarrow$										
Yoo et al. 2019 [67]	Assistance	10	I/C	Ch	$\uparrow$		$\uparrow$	$\uparrow$								
Cappello et al. 2018 [51]	Assistance	9	–	Sb/Ch	$\uparrow$											
Zhou et al. 2019 [52]	Assistance	3	–	Ch		$\bullet$			$\bullet$							
Nazari et al. 2021 [65]	Assistance	2	C	Ch												$\bullet$
Kang et al. 2018 [55]	Assistance	2	–	–												$\bullet$
Randazzo et al. 2018 [68]	Assistance	2	C	Ch												$\bullet$
Bützer et al. 2021 [64]	Assistance	1	–	Ch								$\bullet$				
Tran et al. 2020 [66]	Assistance	1	–	–		$\bullet$			$\bullet$							
Kim et al. 2019 [40]	Assistance	1	–	–												$\bullet$
In et al. 2015 [54]	Assistance	1	–	Ch												$\bullet$
*FES*																
Harvey et al. 2016 [76]	Therapy	70	I/C	Sb				$\downarrow$				$\downarrow$				
Thorsen et al. 2013 [74]	Assist./Therapy	27	I/C	Ch								$\uparrow$				
Popovic et al. 2011 [11] (or Kapadia et al. 2011 [46])	Therapy	21	I	Sb	$\uparrow$		$\uparrow$	$\uparrow$								
Scott et al. 2018 [48]	Therapy	14	I/C	Ch			$\bullet$									
Kilgore et al. 2018 [19]	Assistance	13	I/C	Ch						$\uparrow$						
Kowalczewski et al. 2011 [78]	Therapy	13	I/C	Ch								$\uparrow$				
Kapadia et al. 2013 [62]	Therapy	8	I	Ch	$\bullet$		$\bullet$	$\bullet$			$\bullet$					
Jovanovic et al. 2021 [63]	Therapy	5	I	Sb	$\uparrow$		$\uparrow$	$\uparrow$			$\uparrow$					
Trincado-Alonso et al. 2017 [75]	Therapy	4	I	Sb/Ch				$\bullet$			$\bullet$					
Martin et al. 2012 [77]	Therapy	3	I/C	Ch		$\uparrow$			$\uparrow$							
Fattal et al. 2022 [69]	Assistance	2	C	Ch											$\bullet$	
Venugopalan et al. 2020 [70]	Assistance	2	C	–					$\bullet$	$\bullet$						
Müller-Putz et al. 2019 [41]	Assistance	2	I/C	Ch						$\bullet$						
Memberg et al. 2014 [59]	Assistance	2	I/C	Ch												$\bullet$
Pedrocchi et al. 2013 [72]	Assistance	2	I	Ch												$\bullet$
Cajigas et al. 2021 [49]	Assistance	1	C	Ch		$\uparrow$		$\downarrow$								
Bockbrader et al. 2019 [20]	Assistance	1	C	Ch					$\downarrow$	$\uparrow$	$\uparrow$	$\uparrow$	$\uparrow$	$\uparrow$		
Annetta et al. 2019 [21]	Assistance	1	C	Ch												$\bullet$
Heald et al. 2019 [61]	Assistance	1	I	Ch						$\bullet$						
Colachis et al. 2018 [58]	Assistance	1	C	Ch						$\uparrow$						
Schwemmer et al. 2018 [57]	Assistance	1	C	Ch						$\uparrow$						
Ajiboye et al. 2017 [60]	Assistance	1	C	Ch												$\bullet$
Bouton et al. 2016 [56]	Assistance	1	C	Ch							$\uparrow$					$\bullet$
Rohm et al. 2013 [71]	Assistance	1	C	Ch						$\bullet$						$\bullet$
Gan et al. 2012 [73]	Assistance	1	–	Ch												$\bullet$

^a^N: sample size; LC: lesion completeness; TSI: time since injury: Ac: acute (TSI<1month), Sb: sub-acute (1 month<TSI<6 months) or Ch: chronic (TSI>6 months). LC is based on the American Spinal Injury Association Impairment Scale (AIS)$\bullet$ means that the study includes the assessment tool, but does not present any statistical analysis.$\uparrow$ (or $\downarrow$) means that the study includes the assessment tool and reports that the intervention/device produced superior (or equivalent/inferior) outcomes when compared to control/without the device assistance. The hyphen means lack of information. TRI-HFT: Toronto Rehabilitation Institute-Hand Function Test; JTHFT: Jebsen-Taylor Hand Function Test; FIM: functional independence measure; SCIM: spinal cord independence measure; BBT: box and block test; GRT: grasp and release test; GRASSP: graded redefined assessment of strength, sensibility and prehension; ARAT: action research arm test; CUE-T: capabilities of upper extremity; QIF-SF: Quadriplegia Index of Function-Short Form; MCS: motor capacities scale; nSTD: non-standardized test

1) TRI-HFT

Toronto Rehabilitation Institute-Hand Function Test (TRI-HFT) [81] is an evaluation tool used to assess improvement in unilateral gross motor hand function. The TRI-HFT consists of 3 components: the first evaluates the power grasp, the lateral pinch, and precision grip, through the manipulation of 10 ADLs objects (max. 7 points per object); the second component uses 9 wooden blocks of various masses and surface finishes (maximum of 7 points per object) to evaluate the strength of both power and lateral grasps; the third part is not validated, thus it will not be considered in the following discussion.

*Assistance.* Comparing the baseline and assisted condition, without any training time, participants in Cappello’s study [51] improved $33\%$ (relative to the maximal test score) in the first component of the test, and $37\%$ in the second. Yoo’s paper [67] reported similar relative improvements, of $22\%$ and $29\%$, for the first and second components. All the results of these two research studies that used soft robots are statistically significant. However, both agreed that their device designs did not allow for manipulation of small objects, such as pencils.

*Therapy.* In an uncontrolled study, Osuagwu and colleagues [50] described a protocol in which the participants were encouraged to practice a set task and perform their usual ADLs using the robotic glove, for a minimum of 4 hours per day, for 12 weeks. A significant effect was observed only for the power grasp component after 6 weeks, participants improved $12\%$ relative to the maximal test score. In a randomized control trial, Popovic and colleagues [11] (or [46]) combined 40h of conventional occupational therapy with 40h of functional electrical stimulation therapy. The intervention group significatively improved $23\%$ and $21\%$, for the power and lateral grasps TRI-HFT components (the control group received 80h of conventional occupational therapy). A similar protocol was reported in [62] but they did not present any statistical analysis due to the small sample size. Although they used different technology, it is worth noting that Osuagwu’s work [50] involved chronic population while Popovic’s study [11] recruited subjects with sub-acute SCI. Furthermore, the first study employed a more intense therapy (mean glove usage of about 120h, at week 6), whereas the second was moderate (80h, after 8 weeks). In a more recent study of the same research group as [11] and [62], Jovanovic and colleagues tested a bilateral rehabilitation protocol using non-invasive BMI controlling a superficial FES system, during (on average) 30 1-hour therapy sessions [63]. The authors used TRI-HFT to assess separately the left and right upper extremities. For the left and right upper extremities, the mean change score on the Object Manipulation component was $38\%$ and $16\%$ (relative to the maximal test score), respectively, comparing baseline to discharge.

2) JTHFT

The Jebsen-Taylor Hand Function Test (JTHFT) [82] is a standardized 7-item test designed to evaluate fine and gross motor hand function using simulated ADLs (writing, simulated page-turning, lifting small objects, simulated feeding, stacking, and lifting large, lightweight, and heavy objects). Time of performance is recorded for each task, thus, shorter times indicate better performance.

*Assistance.* Tran and colleagues [66] reported that JTHFT performance was worse when the subject wore the exoskeleton. These results were attributed to the delays of the voice control system and motors. Zhou’s study [52] showed (without any statistical analysis) lower average times for some participants when using the device, however there was a high variability in these responses and some participants took longer when using the device. Correia and colleagues [53] did not find any statistically significant changes in mean completion time when using their device. Both [53] and [52] hypothesized that the low performance was due to lack of participant’s training and adaptation time. Correia and colleagues [53] also measured completion rate, which was calculated by the ratio between the items completed by the participant and the total number of items in a certain JTHFT subtest. In this case they found that participants improved from a median completion rate lower than $30\%$ at baseline, to $76\%$, with the active glove. Controlled by a ECoG device implantation and actuated by superficial electrical stimulation, the subject in [49] significantly improved performance when lifting small objects, lifting light cans and lifting heavy cans. The authors also reported increase in the handwriting clarity while wearing the device.

*Therapy.* In a prospective case series, Martin and colleagues [77] administered JTHFT at baseline, immediately after the first session and after 24h of the eight sessions of intervention. For two weeks, participants attended eight 30-min sessions in which electrical stimulation was used to assist a grasp and release task. End-users significantly improved their performance (reducing task time) in $33\%$ (immediately after the first session) and $53\%$ (after 24h of the eighth sessions), both compared to the time spent at baseline.

3) FIM

The Functional Independence Measure (FIM) [83] assesses functional ability in 6 areas (self-care, sphincter management, transfers, locomotion, communication, and social cognition). As the present review focused on the hand function, only the self-care subscale was considered. This subscale is composed by 6 items (eating, grooming, bathing, dressing upper body, dressing lower body and toileting), each one is graded from 0 to 7.

*Assistance.* The participants that used the device introduced by Yoo and colleagues [67] showed a significant increase in FIM score of $18\%$. However, the only significant increase was found in the eating category.

*Therapy.* In the randomized control trial of Popovic and colleagues [11] (or [46]), the combination of conventional occupational therapy (40h) with FES therapy (40h) resulted in $71\%$ significant increase in the FIM self-care subscale, when compared to baseline. Furthermore, after 8 weeks, the self-care subscore of the intervention group was $58\%$ greater than the control (which received 80h of conventional occupational therapy). In [62], a similar randomized control trial compared the FIM self-care scores of two groups that received 39h of therapy, one with FES (intervention) and the other 39h of conventional occupational therapy (control). The authors did not present any statistical analysis but, the control group got a lower subscore when compared to the intervention group. In a more recent study of the same research group, Jovanovic and colleagues reported a significative increase of about $98\%$ in the mean score on the FIM self-care sub-component at discharge, when compared to baseline [63]. In this study, the patients attended on average to 30 1-hour sessions of treatment.

In [48], after a 6-week intervention using their device the authors reported that “no worsening of FIM score was noted”.

4) SCIM

The Spinal Cord Independence Measure (SCIM) [84] addresses three groups of functions in people with spinal cord injuries (SCI): self-care (feeding, grooming, bathing, and dressing), respiration and sphincter management, and a subject’s mobility abilities (bed and transfers and indoors/outdoors). SCIM III is the newest version, created to consider intercultural differences of subjects. In this review, only the self-care subscale was considered (of either SCIM or SCIM III), which score ranges from 0 to 20.

*Assistance.* In Yoo and colleagues’ study [67], no significant increase was observed in any scores of each individual ADLs task. However, the total score significantly increased $32\%$, comparing the assisted and the unassisted conditions. In [49] there was no change in the SCIM score compared to the baseline.

*Therapy.* In Popovic and colleagues [11] (or [46]), following the protocol described before, the study reported that, after eight weeks of treatment, the SCIM self-care subscale score of the first group was $89\%$ superior to the second group. The results obtained by Harvey and colleagues [76] indicate that the addition of a hand training program involving FES to a combination of usual care plus three 15-minute sessions per week of one-to-one hand therapy, did not improve hand function in terms of the SCIM (self-care subscale). Both studies only recruited people living with sub-acute SCI, but Harvey’s sample size was considerably larger. Harvey and colleagues argued that their results may have been associated with the sessions of individualised one-to-one hand therapy and usual care that both groups received. According to them, these treatments may have hindered the effect of FES-based hand training that the experimental group received.

In [63]—a more recent study from the same research group as [11]—the authors reported that three, out of five patients significantly improved in SCIM self-care subscores, exceeding the minimal clinically important difference, after about 30 1-hour sessions of treatment.

Other two studies, [62] and [75], reported SCIM scores, both without any statistical analysis due to a very small sample size. In [62], a randomized control trial was performed comparing the performance of two groups: with 39h of conventional occupational therapy or 39h of FES therapy. After 13 to 16 weeks of treatment, the SCIM self-care subscore of the intervention group (FES therapy) was $46\%$ higher than the one obtained by the control group (conventional occupational therapy). However, the study did not present any statistical analysis. In [75], the effect of FES system was assessed after five sessions of one hour each. However, the results were inconclusive: one subject improved his score, one got worse and the other two obtained the same scores before and after the intervention.

5) BBT

The Box and Block Test (BBT) [85] is a quick, simple and inexpensive assessment tool that measures unilateral gross hand function. It requires the participant to move wooden blocks, one by one, across a partition in the middle of a wooden box. The score is based on the number of blocks moved in 60 seconds. Each hand is evaluated separately.

*Assistance.* In Zhou and colleagues [52], two of three participants scored higher in BBT when supported by the robotic glove without previous training. Comparing the best active condition trial to baseline, one participant improved from 0 to 4 and the other from 7 to 9 blocks. No statistical analysis was presented. Tran and colleagues [66] compared the BBT performance of one subject, with and without a robotic glove. Due to the time delays introduced by the voice control system and motors, the user was able to transfer two times more blocks without the device support. Bockbrader and colleagues [20] used the BBT to test the performance of one subject controlling a surface FES system through an implanted BMI. They concluded that the participant reached a higher rate at baseline using his residual hand function (12 blocks/min) than when he was supported by the BMI-FES (9 blocks/min). In [70], the authors were not clear about the results of BBT they obtained.

*Therapy.* In the prospective case series reported by Martin and colleagues [77], three participants attended, for two weeks, eight 30-min electrical stimulation sessions to assist a grasp and release task. The motor function was assessed with BBT at baseline, after the first session and 24h after the eighth session of intervention. There was only a significant difference between the baseline (mean of 18 blocks) and the post-8 session (mean of 24.67 blocks), but not immediately after the first session.

6) GRT

The Grasp and Release Test (GRT) [86] is designed to evaluate neuroprosthetics performance in individuals living with SCI. Using palmar or lateral grasp, the participant is required to pick up, move and release six objects of different sizes, weights and textures (peg, block, video tape, fork, can and paperweight). The aim is to release the objects in the target region as many times as possible in 30 seconds. Successful transfers are recorded.

*Assistance.* In [20, 58] and [57] the same system (BCI-FES) was tested with the same subject, but at different times. Accordingly, they presented similar results in terms of GRT assessment. In [58] and [20], the BCI-FES significantly improved median success rates for all the objects, except the block—which usually, required tip-to-tip grasp type. Results in [57] also indicated improvements using the BCI-FES although they only tested three objects (peg, fork, can). Testing an implanted FES system, Kilgore and colleagues [19] reported that, prior to surgery, the majority of participants could manipulate at least two, out of the six GRT objects. After the surgery, with the system turned on, this number increased to five. The heaviest objects resulted in more failures to manipulate the object. Heald and colleagues [61] tested a similar implanted system, but controlled by a different method, with only one subject. The authors only reported successful trials, instead of the number of blocks transferred within the test time. At baseline (without any supporting system), the subject could only transfer the peg and the block, but with the FES system, all the six objects could be successfully manipulated. In [70], the authors did not compare the GRT scores with and without wearing the FES system, but assessed the participants once week during a 12-week long clinical study, always using the device, to test their ability to learn how to use the neuroprostheses. The two participants increased their GRT scores in 80$\%$ and 142$\%$, after 4 and 6 weeks, respectively. In Rohm and colleagues’ study [71], although the GRT was mentioned (with the same main reference), they only described the transfer of “single blocks”, “double blocks” and pegs. They reported that the subject succeeded in manipulating these objects in 17 out of 26 trials. Similarly, in [41] the authors only included three objects in the test (namely, paperweight, videotape and cylinder) and both patients performed better when wearing the system compared to their baseline condition.

*Therapy.* None of the included papers used the GRT to evaluate the therapeutic effect of a SR or FES device.

7) GRASSP

The Graded Redefined Assessment of Strength, Sensibility and Prehension (GRASSP) [87] is a standardized test developed to assess three major domains of hand function: strength (max. 50 points), gross grasping ability (qualitative prehension, with max. of 12 points), prehensile skills (quantitative prehension, with max. of 30 points) and sensibility. Due to the focus of this paper, the sensibility domain will not be considered.

*Assistance.* In [56] and [20], the same FES system was tested with the same subject, reporting similar results. The authors normalized the GRASSP domain scores to benchmarks of the International Standards for Neurological Classification of Spinal Cord Injury (ISNCSCI) and the American Spinal Injury Association Impairment Scale (ASIA) [88]. Thus, Bouton and colleagues [56] reported that when the participant used the implanted BMI, his strength improved from C6 to C7-C8 level, his gross grasping ability improved from C7-C8 to C8-T1 level, and his prehensile skills improved from C5 to C6 level.

*Therapy.* Without showing any statistical analysis, Kapadia and colleagues [62] reported that, after 13 to 16 weeks, all GRASSP components in the treatment group (39h of superficial FES therapy) showed a greater increase (from baseline to post-treatment) when compared to the control group (39h of conventional occupational therapy). In a more recent study of the same research group as [62], Jovanovic and colleagues reported significative increase in the strength components in the three participants that completed discharge, after completing 30 1-hour sessions (average) [63].In [75], Trincado-Alonso and colleagues also did not present any statistical analysis but, in this case, they reported inconclusive results based on GRASSP scores.

8) ARAT

The Action Research Arm Test (ARAT) [89] is an assessment tool of upper extremity performance, composed of 19 items, categorized as grasp, grip, pinch, and gross movement. Functional tasks are, for example, lifting and moving blocks of various sizes, pouring water, picking up, and placing small objects. Task performance is rated on a 4-point scale, thus the total score varies from 0 to 57. A positive score change exceeding 5.7 is considered clinically relevant.

*Assistance.* Bockbrader and colleagues [20] used an invasive BMI to control a superficial FES and tested it with one subject. With the system turned on, the total ARAT score significantly increased from 18 to 30 ($32\%$ to $53\%$ of maximum ARAT score). Thorsen and colleagues [74] tested a superficial FES system controlled by EMG and analysed immediate and therapeutic effects. In terms of immediate effects (measured at baseline, comparing the ARAT performance between the conditions with and without the system support), in average, subjects significantly increased only 2 points in ARAT score. Although the authors did not present any statistical analysis, in [64] the ARAT scores also exceeded clinically relevant improvement when the subject was assisted by the ETHZ RELab tenoexo.

*Therapy.* Thorsen and colleagues [74] measured the therapeutic effects of an FES system, assessing ARAT scores without the system at baseline and after 12 2h-sessions of training. Only $11\%$ of the end-users exceeded the clinically relevant change of 5.7 points. The combined effect (i.e., measured without the system, at baseline and, with the system, after the training intervention) showed that $30\%$ of participants exceeded the clinical relevance threshold. With a greater sample size, Harvey and colleagues [76] suggested that the addition of a hand training program involving FES to a combination of usual care plus three 15-minute sessions per week of one-to-one hand therapy, did not significantly improve ARAT score. According to the authors, the sessions of individualised hand therapy and usual care may have hidden the effects of FES-based hand training. In [78] two different exercise therapies were compared, both including FES and delivered by in-home tele-therapy. One of the exercise therapies was referred as being conventional and the other used a specific workstation for the exercises and a modern FES device. The conventional therapy did not increase the ARAT score above the clinically relevant threshold, while the other reached 7.41 points of significant improvement.

9) CUE-T

The Capabilities of Upper Extremity (CUE) [90] assessment comprises thirty-two activities (categorized by, reaching and lifting, pushing and pulling, wrist actions, hand and finger actions) that are scored based on participant self-report and physical or occupational therapist observation, from 0 to 4 (unable, severe difficulty, moderate difficulty, mild difficulty, no difficulty). The maximum unilateral (arm + hand) converted score is 60.

*Assistance.* Only one study used CUE-T assessment. Bockbrader and colleagues [20] reported that the unilateral total score increased from 27 to 49 ($45\%$ to $82\%$ of maximum CUE-T score) when comparing assisted and unassisted conditions. The system could not improve reaching and lifting or pushing and pulling scores.

*Therapy.* None of the included papers used the CUE test to evaluate the therapeutic effect of a SR or FES device.

10) QIF-SF

The Quadriplegia Index of Function-Short Form (QIF-SF) [91] is based on a self-rating interview and assesses the independence level of people with SCI according to 6 self-care tasks.

*Assistance.* Only Bockbrader and colleagues [20] used the QIF-SF assessment. The authors compared the actual functional independence rated at the participant’s home and his expected level of function to use the system at home. The user reported expected to gain “independence with assistive device” for grooming, feeding, and patient-lift transfers, which were not mentioned to being done before trying the system.

*Therapy.* None of the included papers used the QIF-SF test to evaluate the therapeutic effect of a SR or FES device.

11) MCS

The Motor Capacities Scale (MCS) [92] is an evaluation tool that was specifically designed and validated to assess arm and hand function of people with SCI that underwent a tendon transfer surgical procedure. The assessment has three sub-categories (A, B, C and D), but only MCS-C and D were considered in this review because they are related to the hands (C, right hand, and D, left hand). The patient needs to perform three steps (grab, hold, and release) with different ADL objects. A score is given to each step using a four-point scale (maximum 72).

*Assistance.* The work of Fattal and colleagues [69] was the only study to use the MCS. The authors presented a system with invasive electrodes controlled by the user’s shoulder position. According to the article, with the system turned off, hand opening and closing was not possible in both subjects that participated in the study. With the stimulation, MCS-C score rose from 18 to 39 and from 18 to 37 in patients 1 and 2, respectively, after 27 days using the device.

*Therapy.* None of the included papers used the MSC test to evaluate the therapeutic effect of a SR or FES device.

12) Non-standardized test

In 12 out of the 37 documents (32$\%$) of the final collection, the authors used non-standardized tests to evaluate the end-user’s performance wearing a FES system or a robotic glove for assistance or their therapeutic effects. In some studies, the non-standardized test consisted of a grasp and release task, picking up and releasing ADLs objects of various shapes, like a plastic bottle, a banana or a baseball [54, 55]. Other authors asked the subjects to execute other ADLs, like eating, writing, drinking a cup of coffee [59, 60, 68, 71] or simulating a real activity [56]. In addition, in one study [21] they designed a very specific activity involving hand supination and pronation. In [73], functional tests were not described but the authors reported that the subject of the study used the implanted device for 37 months to support ADLs. In [72] a non-standardized 3-level score evaluation was performed by three experts. Finally, some authors used only non-standardized tests [21, 40, 54, 60, 65, 68, 72, 73], others combined it with an assessment of impairment (e.g., range of motion or strength measurements) [55, 59], and others mixed up with standardized functional assessment tools, such as GRT [71] and GRASSP [56].

### Discussion

The recovery of arm and hand function is one of the main therapeutical priorities of people with tetraplegia [2–4]. Soft Robotics (SR) and Functional Electrical Stimulation (FES) are two technologies that may either assist movements during daily tasks or accompany the conventional intervention in protocols for hand function therapy. Although devices based on SR or FES are not novel, they still demand technological development to be fully integrated into the end-user’s routine and, most importantly, they need to be clinically validated with a view to represent a safe, reliable and effective treatment for hand function after SCI. To this extent, the present review compiled the available articles that reported functional outcomes obtained through the use of SR or FES devices (including their combination), assisting or treating hand function of people living with SCI.

**Fig. 3:**
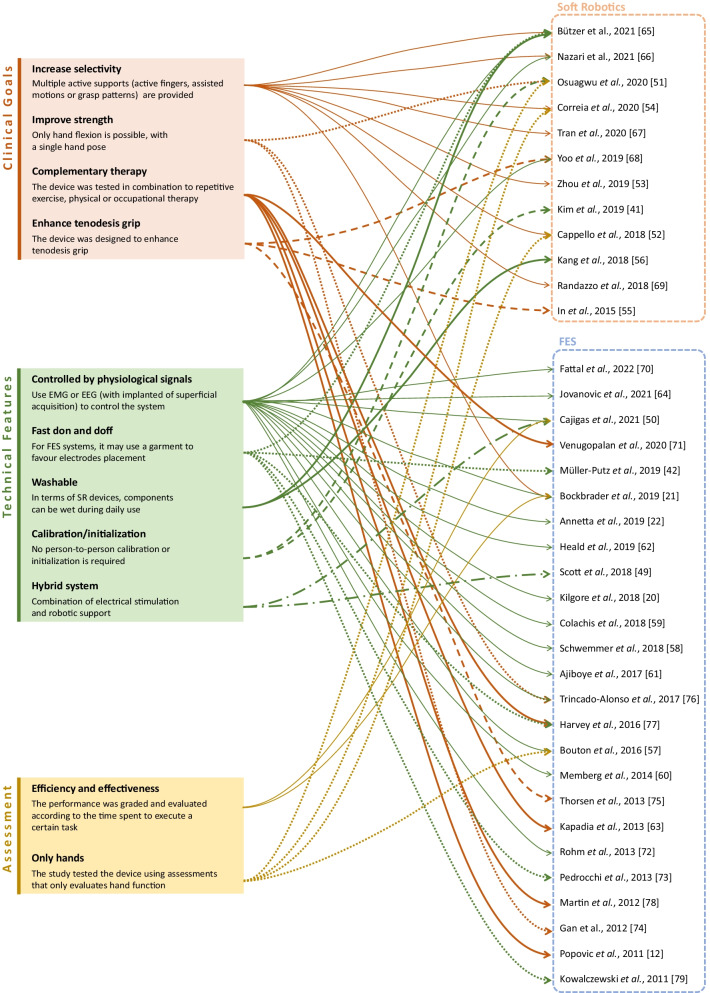
Different domains (clinical goals, technical features and assessment tools) and their connections to the studies of the final collection

The final collection included a total of 37 articles, 12 SR and 25 FES studies. They focused on different clinical goals, tested devices with various technical features and employed multiple assessment tools to evaluate functional outcomes. Figure 3 connects the selected studies with each of these domains.

Following, key-points and recommendations are listed together with the discussion of each point.

*Key-point 1:* Most studies have a system-centric approach and are limited to research prototypes.

*Recommendations:* Increase the focus on functional outcomes. We suggest the use of other commercially available devices designed for different clinical populations, such as stroke. 

Most studies about restoring hand function in people with SCI, using SR or FES technology, have a system-centric point-of-view, which means the articles usually evaluate the performance of the engineered system but fall short in evaluating the clinical added value and testing with end-users. As a result, there are scarce evidence about functional outcomes produced by this sort of devices, with this specific population. Indeed, 18 out of the 37 studies included in our final collection were published by only five research groups. Additionally, most of the reported systems are still research prototypes, which usually have limited feasibility in terms of clinical testing. This is a limitation of the present review, as it only included published articles from year 2010 onwards, which may represent a very short time for research to get translated into clinical practice. FES devices have been extensively used in clinical settings for the last two decades and commercial devices are currently available. Even though, most studies combined commercial stimulators with customized setups (e.g., to change the system to detect user intention), which makes the entire system as experimental as any laboratory prototype. In terms of SR, although its use as rehabilitation tool is relatively new, there are products originally designed for other clinical conditions (e.g., stroke) that could be tested for people with SCI. According to the present review, only one study reported functional outcomes following this approach. We believe that similar studies should be pursued in a way to validate devices that could have a prompt clinical application for people with SCI.

*Key-point 2:* Certain technological components of FES and SR related to portability and easy-to-use capabilities must be improved. The resources should be better prescribed to match end-user’ needs.

*Recommendations:* From an engineering perspective, further work is needed to produce an easy-to-use, intuitive and fast-response user intent detection method. Portability, donning/doffing and the time spent with calibration are other major technological challenges. Innovative devices should embrace a feedback system (either visual or haptic) and a system for continuous control the movements. For clinicians, the ideal selectivity level, active support and even the user intent detection method should be determined according to the individual situation of each end-user. Further investigation is necessary to match these features to different subject’s conditions.

The fact that most devices described in this study were at an early stage of development translates to technological and usability issues that need further investigation. For example, there is not a consensus regarding the ideal user intent detection method for a given user level of impairment. Some devices use buttons to control the state of the glove or to trigger the stimulation, which is a reliable and robust method to detect user intent, but this system does not provide the user with intuitive interaction and requires the support of the contralateral hand, which may restricts the usage in the target population of people with SCI and limited hand function bilaterally. Other studies use an intuitive and unobtrusive method to operate the system, such as, the brain machine interfaces (BMI), either via non-invasive EEG electrodes or brain implantation. In [60], for example, the intracortical implant was used to control the FES stimulation and the subject reported to make movements without the need to concentrate hard at the task. At the cost of a surgical procedure, the invasive system provides more accurate data when compared to the superficial one, thus it allows to recognize and select between different grasp patterns and could also control continuous movements [93]. Other studies have proposed alternative methods to detect user intent, such as voice control, superficial EMG, force sensors, but their successful applicability depends on specific condition of the user. To resolve this discrepancy, this matter requires a user-centered approach to identify the most appropriate detection method based on the users’ individual residual function and needs. The study of Predrocchi and colleagues [72] was the only one in our collection that used a user-centered approach to determine the best configuration of the system to match the patient’s needs, varying the intention detection method (between button, eye tracking or BCI) and the actuation system (either FES or a robotic orthosis).

Other technological challenges identified among the selected devices were the time delay introduced by the control systems, the frequent calibration to recognize user intent and the difficulty to independently don and doff the glove or place the surface electrodes without assistance. Additionally, interactive and constant feedback should be considered in order to enhance the practice-induced brain and spinal plasticity [14, 94] and increase usability features. None of the studies reported a feedback system, except a visual interface used to support the training time. In terms of challenges from the FES selected studies, all devices, except one, were reported to use a biphasic waveform, and most of them did not vary the pulse amplitude or the frequency. In a recent study with people with stroke, authors reported different outcomes for neuromuscular electrical stimulation of varied frequency [95].

Related to the SR collection, there is a need to improve the physical structure of some robotic gloves or hand orthotics. Due to their size and design, part of the fingertips and palm are covered, which hinders the natural user’s somatosensation. People with incomplete SCI may have preserved some sense of touch, thus for those individuals it would be relevant to sense the external objects and tools during manipulation. When the device uses a glove interface [51–54] the entire hand is covered, but other designs preserve the full area of fingertips [67] or have no obstructions on palms and fingertips [64, 65, 68]. Interestingly, either a glove with absent somatosensation [51] or a hand orthosis with no obstruction at the fingertips [67], can present difficulties for end-users to manipulate small objects (like pencils), which represent another issue that should be addressed.

In terms of the active support provided by SR or FES devices, some of them only consider finger flexion, thus the manipulated objects are released when the system is not activated (relax state) [50, 67, 74, 75]. This method may be useful, for example, to enhance tenodesis grip [67, 74], and considerably reduces system complexity, which means less actuators or few number of electrodes, as well as smaller size and lighter weight. Although a stable and secure finger flexion may be enough to match some end-users’ needs, it is worth expanding the potential active support of SR and FES devices since most ADLs rely on an extensive grasp taxonomy [80, 96, 97]. In this respect, among FES articles, the studies involving a BMI interface and a multi-pad stimulation system, presented the widest range of grasping types, including precision and power grasps [20, 21, 56–58]. The use of a matrix with multiple electrodes seems to be a promising way to balance between precision of movement and invasiveness. In the SR collection, it is plain that a broader set of hand patterns is possible with more active fingers and multiple assisted motions (e.g., the lateral key depends on thumb adduction and abduction), even if some motions are assisted passively [64].

The user intent detection method also plays a crucial role in the grasping performance. For example, the system presented by [20, 21, 49, 56–58] used superficial stimulation and invasive cortical electrodes to detect user intention of movement, whereas studies [19, 59, 61] employed implanted electrodes to stimulate nerves and muscles, and implanted EMG for the user intent detection. Although invasive stimulation is known to have higher resolution, which means it is suitable to produce complex hand poses, the studies in [19, 59, 61] presented inferior performance in terms of grasping types when compared to the system presented by [20, 21, 56–58]. In this case, the intracortical implant seemed to be more appropriate to recognize and select different hand poses than the EMG technique. Hence, the system described by [60] was potentially the most powerful among our collection since it combined percutaneous electrical stimulation and intracortical signals. Unfortunately, the authors did not elaborate at the grasp taxonomy, as much as the studies in [58] or [20] did. Still, in terms of innovative user intent detection, it would be useful to expand a new paradigm of continuous control interface, instead of the current situation in which a variety of hand patterns options is offered to the user. This would allow the end-user to combine familiar movements to compose novel hand functions, following a process similar to the generalization, which is typical in human motor learning.

A semi-custom approach—off-the-shelf items that best fit user’s needs combined with customizable protocols, assisted and monitored by the wearable devices—may be suitable in order to ensure the right fit for different subject’s conditions and the available SR and FES technological resources.

*Key-point 3:* Devices are designed and tested with a specific purpose, either assistance or therapy, but not both.

*Recommendations:* Design and test devices for both assistive and therapeutic purposes.

In a research involving elderly, subjects with stroke and healthcare professionals, Radder and colleagues pointed that the end-users prefer to have wearable robotic devices not only for assistance during ADLs, but also for therapeutic goals [98]. This suggestion of unified systems has been identified in a recent literature review about SR for hands [27] and has also already been tested for other neurological conditions, such as stroke [10]. Almost all devices we identified were described by the authors as either assistive or therapeutic tools for people with SCI, although most of them could be utilised in both ways after minor or any changes. Among the studies of our collection, only one SR [50], and two FES studies [76, 78], assessed the therapeutic effects of a device originally designed for assistance. Particularly, in [74], Thorsen and colleagues evaluated both the therapeutic and the assistance effects of a myoelectrically controlled functional electrical stimulator. As expected, the greater clinical relevance was observed for the combination of training and assistive effects.

This characterization between either assistive or therapeutic goals, may be partially motivated by the differences in the validation process and clinical testing of both systems. The former usually undergoes an observational study, comparing the same hand function tasks with and without assistance, whereas the therapeutic effects are commonly investigated with longitudinal studies, assessing the same motor functions, without wearing the system, before and after a certain training time. In studies in which the user acceptance is assessed, different aspects are evaluated, such as the subject’s perception after wearing a device for a full day or by using the device during clinical sessions. Additionally, assistive devices prioritise the individual aspect and the cost affordability, whereas the therapeutic tools prioritises the shared use of the device by different users. In despite of these differences, SR and FES systems should be designed and validated for assistive and therapeutic purposes in order to accomplish integral recovery of hand function and integrate it back to the end-user’s routine. A system with unified goals may help increases device usage time as being supported during ADLs, and consequently the therapeutic outcomes are enhanced due to the increased dosage (according to the practice-induced neural plasticity mechanism [14, 33]). Additionally, systems combining assistance and therapeutic aims could lead to customizable and adaptative rehabilitation programs, supported and monitored by SR and FES wearable devices.

Nevertheless, to be integrated to the daily basis, a system must be portable and allow the user to independently don and doff, among other desirable features. To date, few studies of our collection described devices ready to be used at home [19, 41, 49, 50, 59, 73, 76, 78].

*Key-point 4:* There is a great variety of assessments used by studies and frequently no standardized tests are adopted.

*Recommendations:* Combine assessments to evaluate functional outcomes in terms of effectiveness and efficiency and focus on the performance of the hands and fingers instead of the entire upper limbs function.

There is no consensus about the preferred functional assessment used to report user’s performance, which limits the comparison between different studies. Our selection identified up to eleven different types of tests, while the SR articles focused on six of them. In terms of the metrics adopted by these assessments, both efficiency or effectiveness can be measured. Efficiency is interpreted as a measure of the efforts expended with the intention of achieving a certain goal and it typically involves timed tasks (e.g., counting the number of successful transfers of wooden blocks within a certain time period). Within our collection of assessments, JTHFT, BBT and GRT fall into this category. On the other hand, effectiveness is associated to the accuracy of the motor execution, thus scores are commonly used to quantify the performance, either based on participant self-report or physiatrist observation. Examples of tests that assesses effectiveness are, TRI-HFT, GRASSP, FIM, SCIM, ARAT, CUE-T, QIF-SF and MCS. Both approaches are interrelated and should be used in a complementary fashion for a comprehensive assessment. Nevertheless, only two studies of our full collection measured the functional outcomes combining both assessment types [20, 49].

Analysing the set of functions each test comprises, it is noticeable that part of them is specifically designed to evaluate hand function (TRI-HFT, GRASSP and JTHFT), while others focus on the performance of ADLs or other tasks that usually involves shoulder and arm function (this is the case of BBT, GRT, FIM, SCIM, ARAT, CUE-T, MCS and QIF-SF). From a user-centric point-of-view, we believe that assessing hand function performance contextualized to the subject’s routine is important, which emphasizes the value of this type of assessment.

However, when isolated, this approach may not represent the current hand function and its improvements could not be perceived due to the low performance of the unsupported limbs. In our collection, eight studies^1^ (all regarding FES) solely used assessments that involve the performance of functions beyond the hands (e.g., arms and shoulders), even testing a neuroprostheses that only acts on the fingers and wrist [19, 48, 57, 58, 61, 71, 76, 78]. This type of assessment may not be appropriate for people with cervical and complete SCI. Thus, we believe that the assessment tool should be based on the current level of function of each patient.

Besides the variability of functional assessment, other factors also limited our examination of functional outcomes. One of them was the use of non-standardized tests to evaluate user’s performance, which happened in almost a third part of the entire collection—with eight studies using no other functional assessment tool [21, 40, 54, 60, 65, 68, 72, 73]. Another limitation we found was the small sample size of most articles, usually designed as case studies or case series. In the case of invasive devices, this may be justified for the low number of eligible participants, but other authors justified their small sample size by a proof-of-concept design. Furthermore, the lack of statistical analysis of some studies also limited the comparison between studies. Considering the twenty-five articles that have used at least one standardized test, eleven of them did not present statistical analysis, and it cannot be directly associated to a limited sample size.

In addition, regarding the details of the analysed population (sample size, lesion completeness and time since injury), our review showed low heterogeneity among studies. The population of most articles was constituted by people with chronic and complete SCI, a small part of papers included subjects with sub-acute SCI [11, 51, 53, 63, 75, 76] and notably none of them considered the acute condition. More importantly, many documents did not describe the participant’s lesion completeness. This is a significant limitation as lesion completeness and level are crucial to determine the most appropriate functional assessment for a certain patient. Additionally, the particular condition description can have an important impact on functional outcomes using these therapeutic or assistive devices [33]. A recent publication involving people with chronic stroke showed that in order to maximize the therapeutic effect of a neurorehabilitation, the treatment program must be in accordance with the severity of each patient’s clinical condition [99]. In this sense, standardized neurological examination may also help to provide context to the observed functional outcomes.

Major functional outcomes of each study were presented to serve as a guideline for clinicians and engineers who are interested in the application and continuing development of these SR and FES technologies, so people with SCI can have access to these technologies and improve their hand function in the clinical and home settings.

## Conclusion

Rehabilitation of hand function plays a crucial role in the independence of people with SCI. SR and FES wearable devices are two promising technologies that can either assist daily tasks or support hand therapy. From an engineering perspective, technological improvements are needed before these devices can be extensively prescribed in clinical setting or for home-based use. For instance, portability, donning/doffing and the time spent with calibration were identified as important limitations of most devices. In addition, an easy-to-use, intuitive and fast-response user intent detection method should be explored further. From a clinician’s point of view, studies should match technological features to end-user’s conditions. Consistent assessment of functional outcomes between studies is another limitation of the studies.

We are confident that SR and FES wearable devices have a huge potential to support hand rehabilitation after SCI. The present narrative review helps engineers providing information on the next steps to develop these technologies and serve as clinical guidelines for clinicians to better prescribe these devices for assistance and therapy of people with SCI.

## Data Availability

Not applicable.

## Abbreviations

SR: Soft robotics
FES: Functional electrical stimulation
SCI: Spinal cord injury
ADLs: Activities of daily living
iBMI and sBMI: Implanted and surface brain machine interface
iEMG and sEMG: Implanted and surface electromyography
TLSS: Three-layered sliding spring mechanism

## Footnotes

1 Although the study [69] only used the MCS test to evaluate functional improvement, the authors solely assessed MCS sub-categories C and D, that are focused on the hands.
